# Supplementary material for: A globally relevant stock of soil nitrogen in the Yedoma permafrost domain
Source: Nat Commun. 2022 Oct 14;13:6074. doi: 10.1038/s41467-022-33794-9 (PMC9568517; doi:10.1038/s41467-022-33794-9)
Supplement: Supplementary file 1 — Supplementary Information [file 41467_2022_33794_MOESM1_ESM.pdf]

Supplementary Information for
"A globally-relevant stock of soil nitrogen in the Yedoma permafrost domain"

by Strauss et al.

Supplementary Methods

Mapping the Yedoma domain

For the areal estimates, we followed Strauss et al.[1] who mapped the core Yedoma domain extent as ~2,587,000 km². This extent is discontinuous due to thermo-erosion and thermokarst processes, and data from local- and regional-scale analyses of Yedoma deposit versus thermokarst-affected areas indicate that ~80% of the Yedoma region area is affected by degradation. The remaining Yedoma deposit (~20%) is then assumed to be ~480,000 km² [1]. We further estimate that ~10% of the Yedoma region is covered with lakes and rivers and thus underlain by unfrozen deposits (258,700 km²) and ~15% is covered with other deposits including deltaic and fluvial sediments (388,000 km²), leaving ~56% (1,460,380 km²) of the Yedoma domain covered by frozen thermokarst deposits in drained thermokarst lakes underlain by Taberite deposits. The areal extent of the Holocene cover was estimated to overlay ⅔ of Yedoma deposits, which is 319,827 km². Because of lacking data, the thickness of Taberite deposits was estimated using the formula:

$$Taberite_{thickness}\ (m) = Yedoma_{thickness}\ (m) - thermokarst_{thickness}\ (m) - \mathrm{EGIT}_{thickness}\ (m) \qquad (1)$$

Equivalent Ground Ice Thickness (EGIT) was taken as an average from the minimum-maximum estimations by Ulrich et al.[2].

Bulk density calculation

For the calculation of bulk density (ρ_b, 10^3 kg m^{-3}), first the volume of the solids (V_s, 10^{-6} m^3) was derived following equation 2.

$$V_s = \frac{m_s}{\rho_s} = \qquad (2)$$

After that, the porosity (n) was calculated following equation 3 using the volume of the pore space (V_p, 10^{-6} m^3) and sediment volume (V_s).

$$n = \frac{V_p}{V_p + V_s} \qquad (3)$$

Finally, we could calculate the bulk density using the negative linear correlation with the sediment porosity (equation 4).

$$\rho_b = (n - 1) \times (\rho_s) \qquad (4)$$

We defined an ice content >20 wt% as the threshold for ice saturation[3]. With this assumption, the absolute ice content allows an estimation of the pore volume. For the determination of ice volume ($V_{ice} = m_{ice}/\rho_{ice}$) an ice density of 0.91 10^3kg m^{-3} was assumed.

Supplementary References

1 Strauss, J. *et al.* Circum-Arctic Map of the Yedoma Permafrost Domain. *Frontiers in Earth Science* **9**, 758360, doi:10.3389/feart.2021.758360 (2021).

2 Ulrich, M., Grosse, G., Strauss, J. & Schirrmeister, L. Quantifying wedge-ice volumes in Yedoma and thermokarst basin deposits. *Permafrost and Periglacial Processes* **25**, 151–161, doi:10.1002/ppp.1810 (2014).

3 Strauss, J., Schirrmeister, L., Wetterich, S., Borchers, A. & Davydov, S. P. Grain-size properties and organic-carbon stock of Yedoma Ice Complex permafrost from the Kolyma lowland, northeastern Siberia. *Global Biogeochemical Cycles* **26**, GB3003, doi:10.1029/2011GB004104 (2012).
